# Early autism detection: a review of emerging technologies, biomarkers, and explainable AI approaches

**DOI:** 10.1186/s13041-025-01269-9

**Published:** 2025-12-25

**Authors:** Rucha Agrawal, Renuka Agrawal

**Affiliations:** Symbiosis Institute of Technology, Symbiosis International (Deemed University), Pune, India

**Keywords:** Autism spectrum disorder, ASD, AI, eXplainable AI, Healthcare, Neurodevelopmental disorder

## Abstract

Autism Spectrum Disorder (ASD) presents as a complicated neurodevelopmental disorder which leads to social communication challenges and repetitive behavioral patterns. Early identification of ASD is crucial to facilitate early intervention that can make a large positive impact on long-term developmental outcomes. With the advent of artificial intelligence (AI) and data-driven diagnoses, there is increased interest in combining machine learning methods with biological and behavioral signatures to detect early ASD. This review provides an overview of broad classes of biomarkers—behavioral, neuroimaging, genetic, and eye gaze—and their respective methodologies, clinical applications, and diagnostic accuracy. For each of these biomarker domains, the research gap has been identified as existing for instance limited interpretability in neuroimaging models, genomics-related ethical and data accessibility issues, and innovation saturation for behavioral measurement. A comparative analysis highlights eye gaze analysis as a promising but under-explored option, providing a balance of cost-effectiveness, non-invasiveness, and potential for real-time, objective measurement. In addition, the application of Explainable AI (XAI) methodologies across these biomarker fields is discussed in order to meet the pressing need for transparency, clinical confidence, and decision-making support. This review makes a final call for further exploration of eye gaze-based models enriched by XAI methods as a future research direction towards filling the gap between algorithmic innovation and real-world, interpretable diagnostics in the context of ASD research.

## Introduction

ASD falls into the category of neurodevelopmental disorders that affect the proper functioning and development of the brain. Such disorders have a wide range of causes, including delicate relationships between genetic, environmental, and occasionally prenatal variables that interfere with proper brain development [1, 2]. Traditionally, the detection and diagnosis of ASD in children have largely been based on clinical evaluations, behavioral observation, and standardized psychometrics [3, 4]. Clinicians apply diagnostic guidelines set in manuals like the Diagnostic and Statistical Manual of Mental Disorders, Fifth Edition (DSM-5) [5] and International Classification of Diseases (ICD-11) [6] to assess developmental delay, intellectual impairments, language deficiency, and atypical social conduct. Our knowledge of these illnesses has advanced as a result of rapid advancements in technology, which have enabled deeper exploration of their underlying biological, cognitive, and behavioral mechanisms [7, 8].

The characteristics of any neurological condition typically first seen in early life are unwillingness to social interaction, communication, & behavior patterns [9, 10]. The complex etiology of such disorders combines several environmental and genetic factors [11, 12]. Early identification of any disorder is important as it enables early therapies to enhance the developmental outcomes and quality of life for such people. However, early diagnosis can be difficult, given the variety of symptoms and differences in how they appear [13–15].

The integration of Artificial Intelligence (AI) in the healthcare domain has transformed diagnostic methods, with better accuracy and efficiency. Machine Learning (ML) and Deep Learning (DL) models are able to process intricate, high-dimensional information, such as electronic health records, neuroimaging, and behavioral tests, to detect subtle patterns that reflect the prevalence of ASD in individuals [16, 17]. Despite this, the opaque character of AI models is a liability in a clinical environment, where the reasoning behind a diagnosis needs to be interpreted. This is where Explainable AI (XAI) becomes invaluable [18–20]. XAI methods, like SHapley Additive exPlanations (SHAP) and Local Interpretable Model-agnostic Explanations (LIME), create transparency by explaining how certain input features drive the model's predictions [21]. Applying XAI methods to data for analysis helps researchers not only find the exact linkages but also identify the particular factors in the dataset affecting these predictions. This method opens new options for precision medicine in ASD by allowing the creation of customized intervention plans that fit an individual's profile [22].

**Fig. 1:**
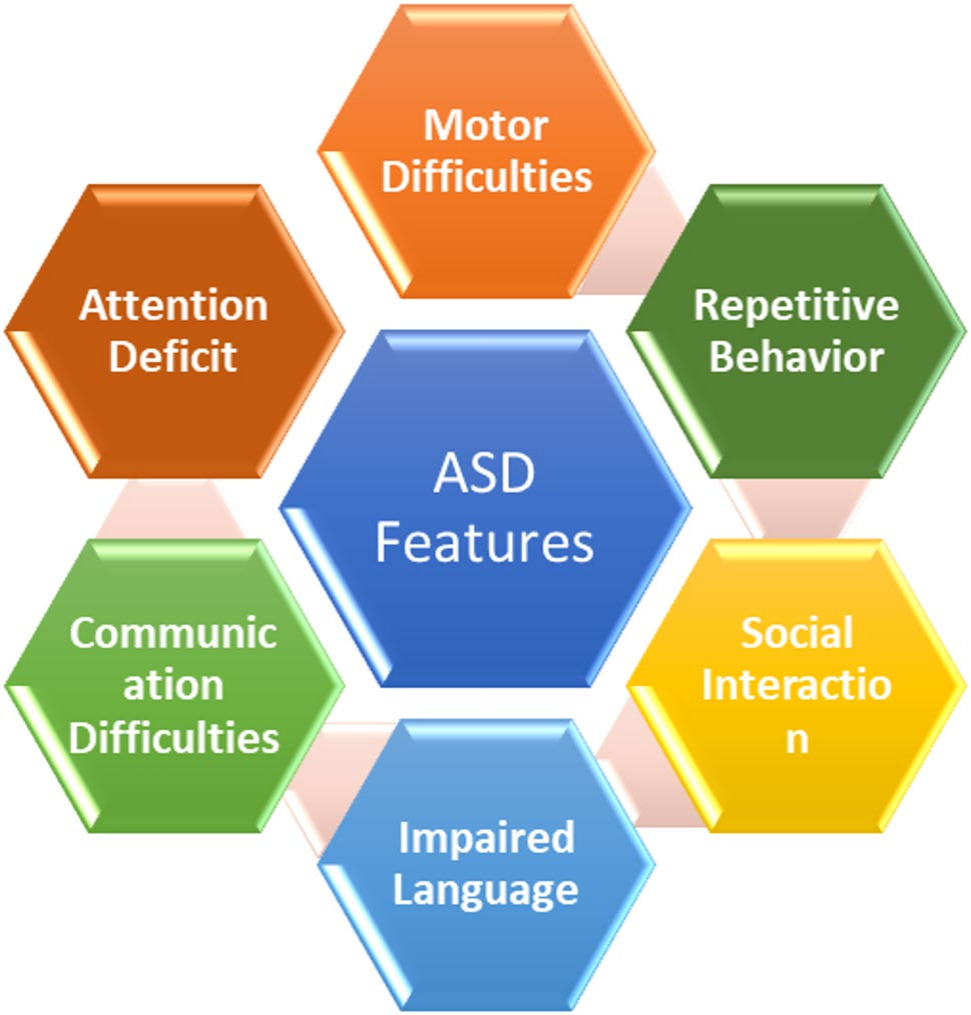
ASD core features

ASD have a heterogeneous etiology and lead to impaired cognition, communication, adaptive behavior, and psychomotor skills [23–25]. As in Fig. 1, a person having ASD may exhibit a variety of behavioral and developmental challenges, such as difficulties in social interaction, restricted or repetitive behaviors, atypical sensory responses, and delays in language or motor development [26, 27]. According to the World Health Organization (WHO), approximately 10–15% of children globally are affected by Neurodevelopmental Disorders (NDD), including ASD, Attention Deficit Hyperactivity Disorder (ADHD), and Intellectual Disability [28, 29]. As of 2024, the Centers for Disease Control and Prevention (CDC) reported that in the United States, 1 in 36 children are diagnosed with ASD. Globally, the prevalence of ADHD in children is estimated to be about 5–7%. In India, studies estimate the prevalence to be 1.6% to 17.9%, depending on the region and diagnostic criteria used. The prevalence of neurodevelopmental difficulties is gradually increasing worldwide as a result of today's more sedentary lifestyles and changing work environments [30, 31]. As a result, early detection is essential for facilitating prompt action and lowering the likelihood of long-term issues.

One of the significant contributions of this study to the literature and practice is its systematic and in-depth analysis of various biomarkers available for the identification, detection, and diagnosis of ASD in children. The study adds value by critically reviewing the implications and roles of few biomarkers for early diagnosis. This review further assesses the scope and possibilities for future research on the biomarkers that have been identified in order to improve clinical outcomes in ASD. Through a structured review methodology, this work provides a comprehensive overview of the present research and identifies gaps, providing clear directions towards future investigation. This review emphasizes the role of AI based biomarker strategies in improving the early diagnosis and intervention of ASD and closing the gap between research developments and real-world clinical applications. By integrating XAI with diverse biomarker modalities, the study underscores the importance of transparency and interpretability in enhancing clinical trust and facilitating informed diagnostic decisions.

The research questions aim to systematically assess the development of AI and XAI techniques for the detection of ASD in children.

RQ1: What types of biomarkers are currently available for the early detection of ASD in children?

RQ2: How do these identified biomarker categories differ in terms of feasibility, cost-effectiveness, diagnostic validity, and interpretability to clinicians?

RQ3: What are the most significant methodological trends and gaps in the research evidence identified in recent studies for each biomarker category?

RQ4: To what extent have models of AI and ML been used to identify ASD based on identified biomarker domains?

RQ5: How can XAI methodologies improve the interpretability and clinical uptake of AI-based diagnostic models for detecting ASD?

Consequently, this paper is a guiding resource for future research exploring the use of AI, XAI techniques in early detection of ASD in children.

This paper is organized as follows: Section “Review methodology” overviews the research methodology. Section “Biomarkers for the diagnosis of ASD” conducts a systematic review of the key biomarkers in ASD detection, with highlights mentioned for each biomarker. Section “Comparative analysis” presents the role of XAI techniques in ASD detection, and the final section emphasizes the challenges and limitations of the study (Table 1).

**Table 1:** Abbreviations

Acronym	Full form
WoS	Web of Science
IEEE Xplore	Institute of Electrical and Electronics Engineers Xplore Digital Library
PRISMA	Preferred Reporting Items for Systematic Reviews and Meta-Analyses
MRI	Magnetic resonance imaging
fMRI	Functional magnetic resonance imaging
QDA	Quadratic discriminant analysis
PCA	Principal component analysis
k-NN	k-nearest neighbors
FL	Federated learning
NLP	Natural language processing
DT	Decision tree
XGB	Extreme gradient boosting
RNN	Recurrent neural network
LSTM	Long short-term memory
Bi-LSTM	Bidirectional long short-term memory
BERT	Bidirectional encoder representations from transformers
BERTweet	BERT model pretrained on Twitter data
DMLRS	Deep machine learning with rule-based SHAP
XGBOOST	eXtreme gradient boosting
DMRCE	Deep models with residual connections and ensemble
ISSA-FS	Improved squirrel search algorithm – feature selection
SVM	Support vector machine
LR	Logistic regression
QT	Quantitative trait
PT	Physical therapy
MAS	Modified Ashworth Scale
AB	Ada boost
LDA	Linear discriminant analysis
RF	Random forest
IGAE	Improved graph autoencoder
GRAE	Graph regularized autoencoder
RFAE	Robust fuzzy autoencoder
CAE	Convolutional autoencoder
RFC	Random forest classifier
DTI	Diffusion tensor imaging
ANOVA	Analysis of variance
UMAP	Uniform manifold approximation and projection
AgHC	Agglomerative hierarchical clustering
CSP	Common spatial pattern
ABIDE	Autism brain imaging data exchange
GNN	Graph neural network
MHACSM	Multi-head attention cross-scale module
RMCN	Residual multi-context network
MSE-GCN	Multi-scale enhanced graph convolutional network
GAM	Generalized additive model
RFECV	Recursive feature elimination with cross-validation
GCN	Graph convolutional network
RFE	Recursive feature elimination
pVAAST	Probabilistic variant annotation, analysis and search tool
MetaSV	Meta structural variation
BMA	Bayesian model averaging
AGP CNV	Autism Genome Project Copy Number Variation
AHC	Agglomerative hierarchical clustering
forecASD	Forecast autism spectrum disorder (Gene prioritization tool)
ET	Eye tracking
PCA	Principal component analysis
TracIn	Influence tracking method (Training data attribution)
EAR	Eye aspect ratio
HOG	Histogram of oriented gradients
ETSP	Eye tracking scan path
ACLNet	Attention-context learning network
STAR-FC	Spatio-temporal attention recurrent fully connected
BrMLP	Branched multi-layer perceptron
DARPA	Defense advanced research projects agency
TCAV	Testing with concept activation vectors
CAM	Class activation map
Grad-CAM	Gradient-weighted class activation mapping
CDSS	Clinical decision support system
EEG	Electroencephalography

## Review methodology

This review adopts a structured approach to evaluate the current landscape of biomarker-based techniques used for the early detection of ASD in children, with a special emphasis on the integration of XAI methods. Literature was sourced from reputable scientific databases including Scopus, WoS, PubMed, IEEE Xplore, and Google Scholar, using a combination of keywords such as “ASD biomarkers,” “Eye gaze autism detection,” “Genomic markers in autism,” “Neuroimaging for ASD,” “Behavioral asd,” and “XAI in ASD detection.” Articles published between 2020 and 2024 were considered to ensure recent advancements were used. Inclusion criteria required that papers be peer-reviewed, written in English, and focus on the use of behavioral, neuroimaging, genomic, or eye gaze biomarkers in ASD diagnosis. Studies unrelated to children, lacking biomarker-based evidence, or not employing AI or XAI methods were excluded. Selected studies were categorized based on the type of biomarker analyzed and the methodological approaches used, with emphasis on diagnostic accuracy, interpretability, feasibility, clinical relevance as well as limitations. Findings were synthesized through thematic analysis and presented using comparative tables and visual summaries to highlight both advancements and gaps in the field.

**Fig. 2:**
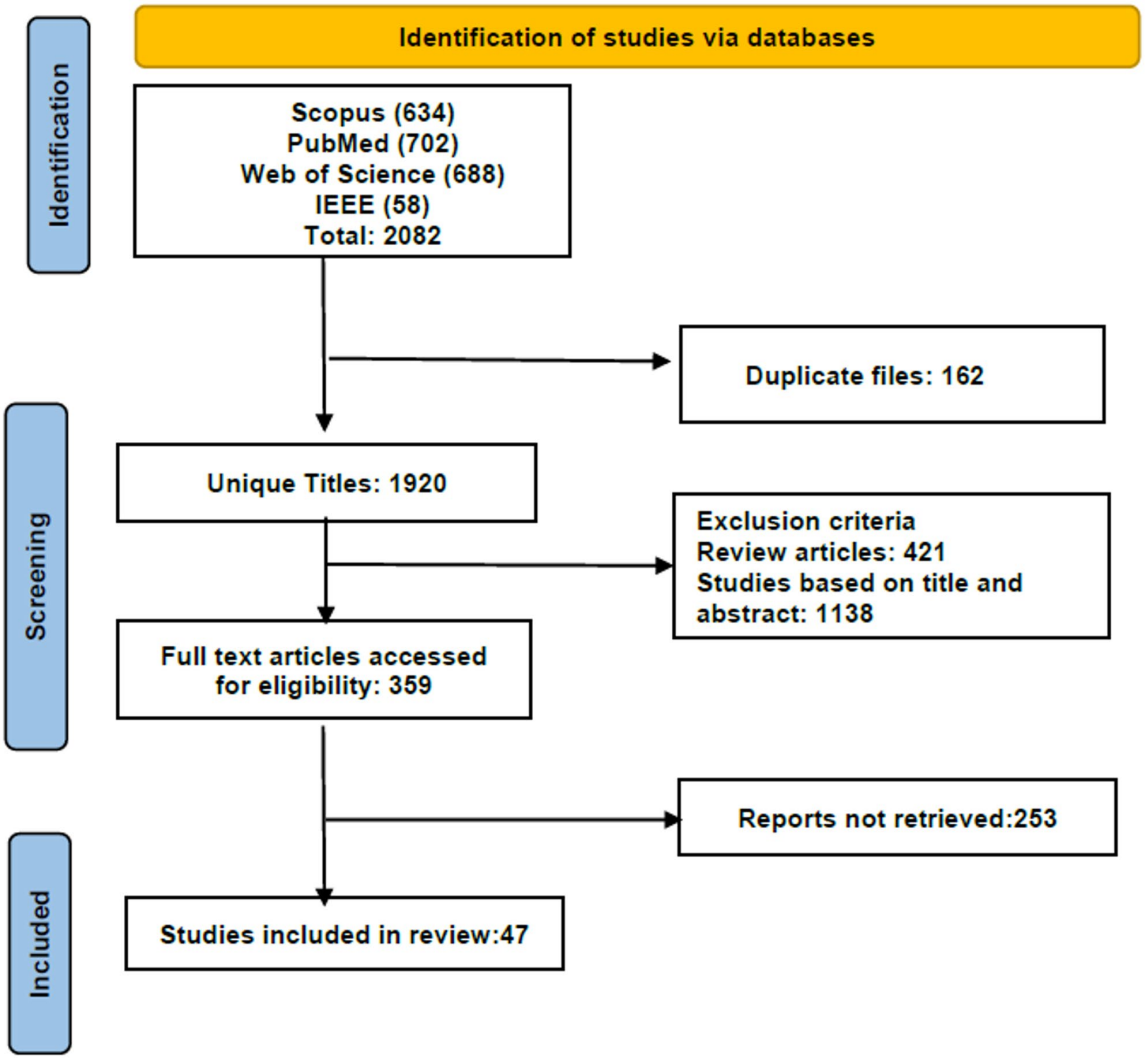
PRISMA model

The PRISMA flow diagram in Fig. 2 illustrates the screening process. The screening involved three stages: identification, screening, and inclusion. Initially, 2082 records were identified. After screening, 1976 records were excluded, resulting in 106 articles in the final review.

**Fig. 3:**
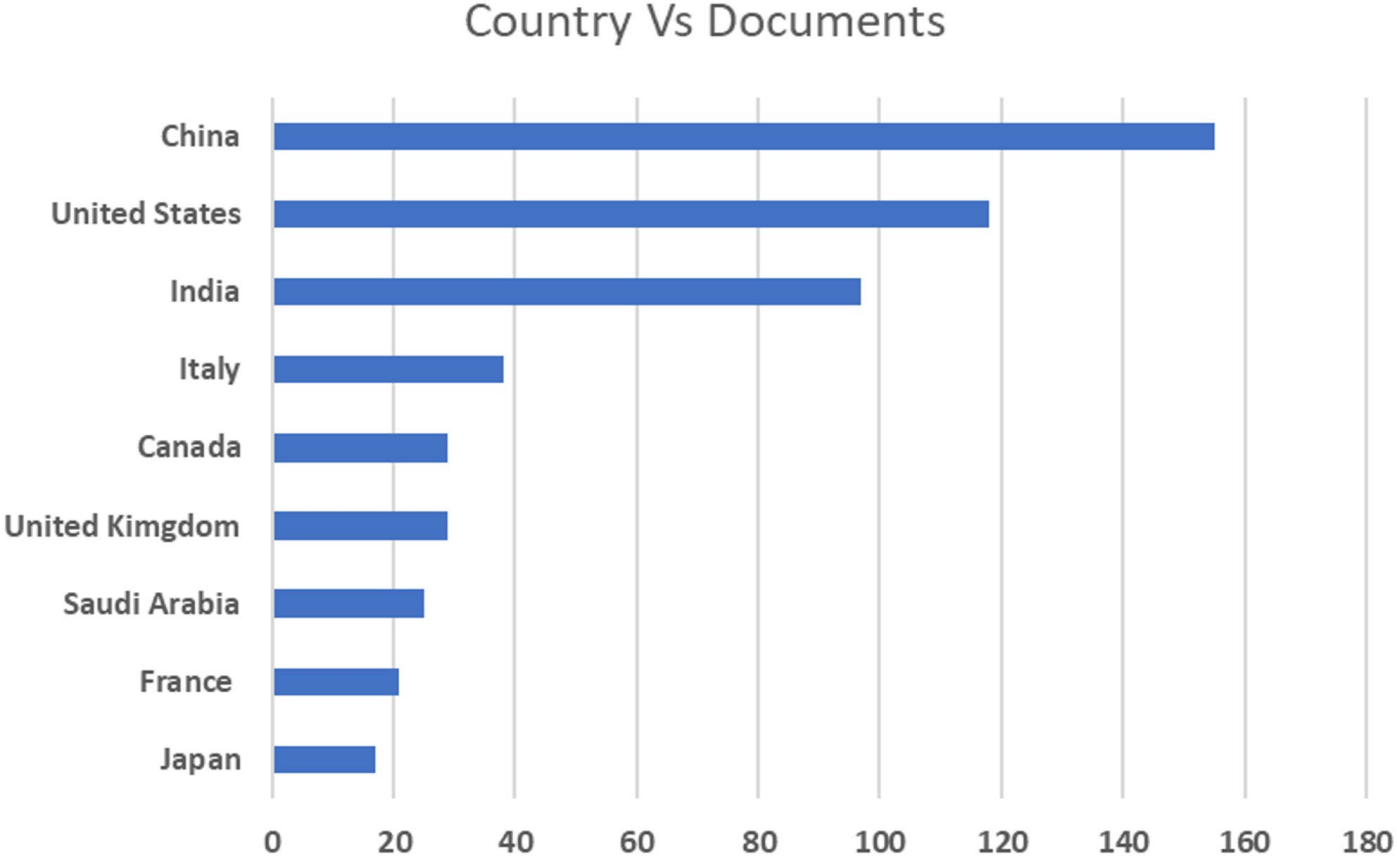
Country-wise ASD detection research output (2020–2025) from Scopus

In light of the increased international interest in the detection of early ASD, however, the available research from India is still relatively modest. An initial search of the Scopus database indicated that there was a disproportionately lower number of publications related to ASD detection methods by Indian research centers, especially those related to biomarker-based strategies and explainable AI. This evident lacuna underscores the urgent necessity for additional region-specific research that is sensitive to local demography, healthcare infrastructure, and diagnostic issues. Spurred by this imbalance, the current research seeks to make a significant contribution to this underdeveloped subject and create advances in the detection of ASD within Indian research. Figure 3 presents a comparative overview of ASD detection research conducted across various countries between 2020 and 2025, based on publication data retrieved from the Scopus database.

## Biomarkers for the diagnosis of ASD

A wide variety of biomarkers is utilized in identifying ASD, each providing distinct information on the underlying biological and functional processes. These biomarkers may be grouped together under categories: Behavioral, Eye Gaze, Neuroimaging, Genetic/Genomic, Speech and Language, Motor and Movement, Physiological, Facial Expression, Metabolic/Proteomic, Microbiome. Together, these biomarkers help improve the accuracy of early diagnosis and facilitate more personalized and effective intervention strategies [32]. This study focuses on four key biomarkers—behavioral analysis, neuroimaging, eye gaze patterns, and genetic markers because these biomarkers have demonstrated the strongest diagnostic potential, the most empirical support, and consistent integration with AI and XAI methodologies in the literature to date. Despite their promise, other biomarkers such as blood-based and metabolic indicators were not included because of a lack of data, inconsistent validation, and a lack of AI-driven research on early ASD detection in children.

**Fig. 4:**
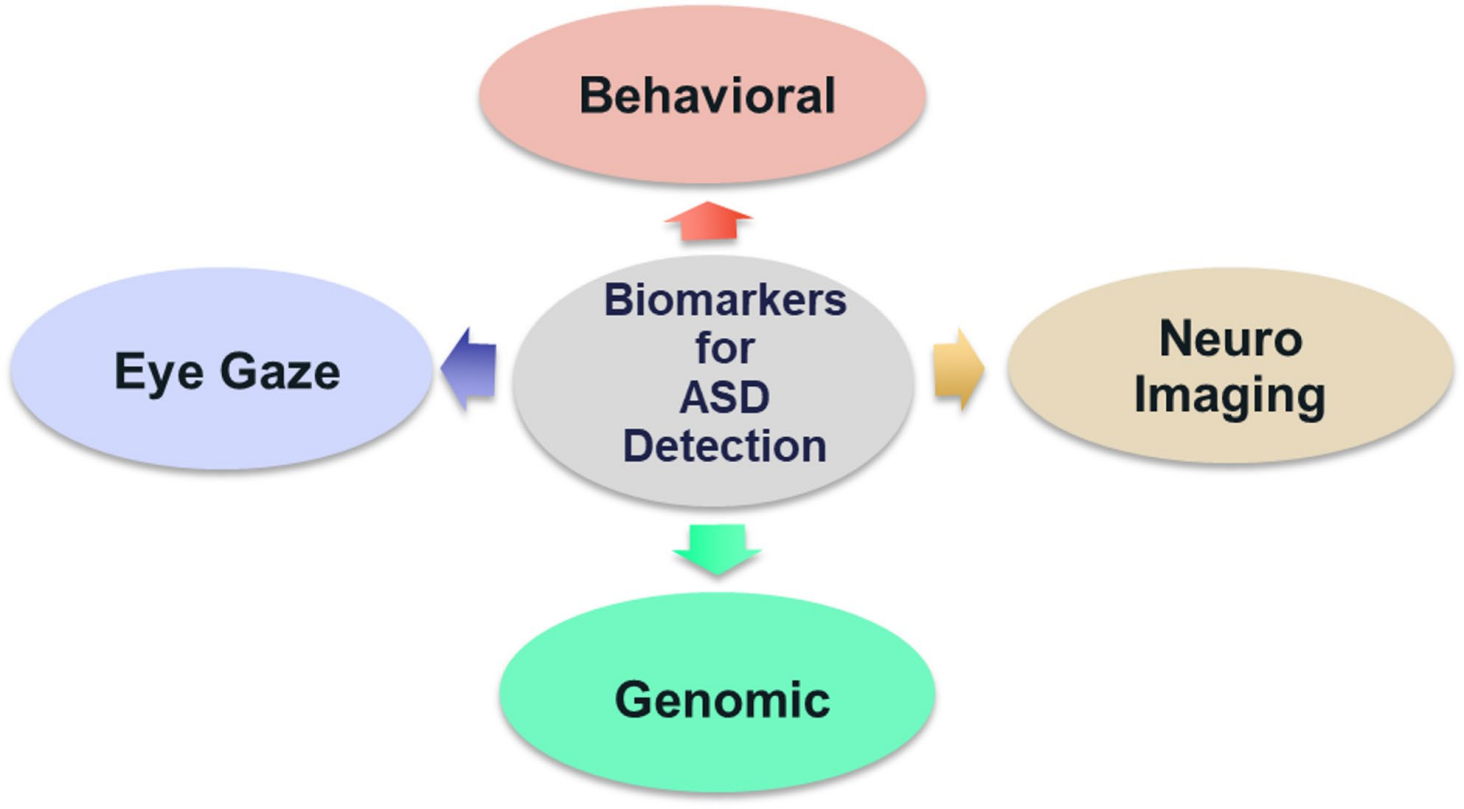
Biomarkers for ASD detection

Assessing social interactions, communication abilities, and repetitive behaviors—all of which are essential markers of disorders like ASD—is part of behavioral analysis. Neuroimaging biomarkers identify structural and functional abnormalities in the brain linked to NDDs using methods like MRI and fMRI. Eye gaze tracking is an objective way to measure changes in visual processing and social attention, which are frequently changed in people with ASD and other NDDs. Certain mutations, copy number variations, and patterns of gene expression associated with certain illnesses can be found using genetic biomarkers. The primary goal of this study is to conduct a detailed review of each major biomarker used in ASD detection, with the aim of evaluating their individual strengths, limitations, and potential for improving the accuracy and reliability of early diagnosis (Fig. 4).

In the following sections, a comprehensive literature survey is presented for each biomarker, exploring its significance, methodologies, and contributions to the early detection and diagnosis of ASD using ML/DL techniques.

### Behavioral analysis

Behavioral analysis has been the primary technique of early detection and diagnosis of ASD in children. It involves the measurement of quantifiable observable traits like repetitive behavior, social withdrawal, communication problems, and sensory sensitivities, which are typical features of the disorder. Such behaviors are typically measured through clinical observation, caregiver report, and standardized screening devices [33]. With advancing artificial intelligence, machine learning algorithms have increasingly been applied to the analysis of behavioral data to improve diagnostic performance, facilitate early intervention, and provide interpretable results that match clinical decision-making. The following Table 2 shows current research done in this area.

**Table 2:** Work done using behavioral analysis for ASD detection

References	Data source	AI/ML method	Outcome/Accuracy	Key insights/Limitations
[34]	Large behavioral datasets (unspecified)	Rule-based classifiers, Decision Trees, Hybrid (with Deep Learning)	Good predictive accuracy (exact values not provided)	High interpretability; Hybrid XAI approach discussed; Limitation in feature selection and deep learning details
[35]	Specialized datasets	Federated Learning with autoencoders, k-NN, MLP, QDA + PCA	Accuracy: 98% (QDA + PCA + MLP in FL setup)	LIME used for explainability; Privacy-preserving techniques integrated; Limitation in robustness & stakeholder validation noted
[36]	404,627 tweets from 252 users	NLP + ML: DT, XGB, KNN + DL: RNN, LSTM, Bi-LSTM, BERT, BERTweet	Accuracy: 88%	Innovative use of Twitter bios; highlights early detection via language; no mention of XAI methods explicitly
[37]	1054 patient samples, 20 behavioral variables	DMLRS (Logistic Regression + SHAP), XGBoost, DMRCE	Accuracy: 99%	Strong behavioral focus; SHAP used for interpretability; lacks cross-population validation
[38]	ASD-Children (292), ASD-Adolescent (104), ASD-Adult (704)	Autoencoder + Butterfly Optimization Algorithm + ISSA-FS	Accuracy: 92%, Precision: 90%, Recall: 93%	Strong age-group variation; advanced optimization; lacks large-scale validation; XAI not directly mentioned
[39]	Home videos submitted by participants	XGBoost.Interpreted with SHAP for feature importance	AUC: 0.938 for the first observer	Highlights early detection of sensorimotor features related to ASD. Limitations: small sample size, male-dominated sample, observer consistency issues
[40]	ABIDE dataset; rs-fMRI with behavioral annotations	SVM, LR, Decision Trees,	Performance strong with DT and RF; exact metrics not reported	Efficient diagnosis via multi-model system; time-consuming feature collection; no XAI usage discussed
[41]	UCI Repository: ASD-Children, ASD-Adolescent, ASD-Adult	Bacterial Foraging Optimization (BFO) for feature selection Whale Optimization Algorithm (WOA) with Deep Belief Network (DBN) for classification and hyperparameter tuning	Accuracy: Child 95.43%, Adolescent: 96.43%, Adult: 95.44%,	The reliance on benchmark datasets may not fully encompass the complexity of real-world clinical scenarios
[42]	ASD screening dataset from Fadi Thabtah (1758 toddlers)	4-layer DL model + SHAP (XAI)	Accuracy: up to 98%;	SHAP used to rank top 7 features contributing 79% to prediction accuracy; strong explainability; real clinical relevance
[43]	Behavioral screening features from QCHAT-10, AQ-10 questionnaires	Feature Scaling (QT, PT, Normalizer, MAS) + 8 ML classifiers AB, LDA, SVM, RF, etc	Accuracy: up to 99.25% (Toddlers with AB); Adults up to 99.03% (LDA)	Feature selection and importance analysis done using IGAE, GRAE, RFAE, CAE; no XAI methods like SHAP or LIME used
[44]	Q-CHAT-10 dataset (1054 toddlers)	LR, NB, SVM, KNN, RFC	LR: 97.15%, SVM: 93.84%, F1 score up to 0.98	Focused on toddlers; no explicit XAI methods; highlighted LR as best performing model
[45]	ASD datasets from UCI repository	Modified Grasshopper Optimization Algorithm (MGOA) + Random Forest	Accuracy: 100% (Child, Adolescent), 99.29% (Adult)	Feature selection via MGOA; No explicit XAI methods; Claimed robust generalization across age groups

#### Highlights of the behavioral analysis for ASD

Substantial research has gone into behavioral analysis for the detection of ASD based on features of repetitive behavior, sensory sensitivity, impairment in communication, and deficit in social interaction. Although explainability and accuracy are high with feature-based models, there are some limitations. Some of the most important gaps are limited data diversity, a lack of clinical validation in the real world, and limited incorporation with other biomarkers. Available behavioral features are mostly used by most research as well, with little room for creativity. A lack of longitudinal analysis and improper utilization of multimodal methods also limit the growth of this research. Also, many people are hesitant to participate in questionnaire-based behavioral analyses, which can limit the reliability and completeness of data collection in neurodevelopmental research. Consequently, while behavioral analysis remains valuable, opportunities for novelty are now limited.

### Neuroimaging analysis

Neuroimaging has emerged as a pivotal tool in the detection and understanding of ASD, providing a quantitative assessment of the structural and functional brain abnormalities in the disorder. Recent advancements have utilized ML and deep learning (DL) algorithms to quantify neuroimaging modalities such as structural MRI (sMRI), functional MRI (fMRI), and diffusion tensor imaging (DTI) to improve the accuracy and validity of ASD diagnosis. sMRI provides high-resolution images of brain anatomy, allowing researchers to examine abnormalities in cortical thickness, brain volume, and gray-white matter differentiation. fMRI measures brain activity by detecting changes in blood oxygenation, enabling the assessment of functional connectivity between different brain regions. DTI, a variant of MRI, maps white matter tracts and is used to assess the integrity of neural pathways, which are often altered in individuals with ASD [46].

Recent advances have integrated these imaging techniques with AI to improve diagnostic precision. ML models trained on neuroimaging data have shown potential in identifying biomarkers associated with ASD, contributing to early and objective diagnosis [47]. These modalities also help in subtyping ASD and understanding individual variability, providing a foundation for personalized interventions [48]. The following Table 3 shows current research done in this area.

**Table 3:** Work done using neuroimaging analysis for ASD detection

References	Data source	AI/ML method	Outcome/Accuracy	Key insights/Limitations
[49]	fNIRS recordings from 51 children (24 ASD, 27 TD), aged 5–7	XGBoost + ANOVA + UMAP + AgHC; Nested Cross-Validation	Accuracy: 96%,	High precision in neurovascular feature detection; limited by small sample size and lack of gold-standard diagnostic tools
[50]	EEG from 10 ASD and 10 TD children (5–7)	Wavelet-based Filter Bank + Regularized CSP + SVM + SHAP	Accuracy: 93.59%,	Identified optimal 12–16 Hz band; SHAP provided feature importance; limited by small, homogenous sample
[51]	ABIDE A (871) & ABIDE B (949); multimodal data (fMRI, aCompCor, metrics, demographics)	DeepASD: Adversarial-regularized multimodal encoder + GNN-based graph classifier	ABIDE A: Accuracy 87.38%, ABIDE B: Accuracy 88.09%	Strong performance across datasets; fMRI most important; lacks fine-grained interpretability; future work may integrate clinical rating scales for validation
[35]	Modified Q-Chat-10 ASD toddler dataset	Federated Learning (FL) + QDA + MLP, Autoencoders + LIME	Accuracy: up to 98%	Combines privacy-preserving FL with XAI; limitations include class imbalance, small dataset, and lack of external validation
[52]	ABIDE dataset	EAG-RS framework with explainable AI for high-order associations	Outperformed BrainNetCNN, BrainGNN, and BrainNetTF	Addressed lack of patient-specific and non-linear connectivity modeling; limited discussion of computational complexity
[53]	ABIDE-I dataset	Cross-Scale + Residual Multi-Context Modules (MHACSM + RMCN)	Avg. Accuracy: 79.7%	Site/scanner agnostic model; varied performance across sites (min 63.3% at SBL), highlighting inconsistency
[54]	ABIDE-I dataset	Multi-Scale Enhanced Graph Convolutional Network (MSE-GCN)	Accuracy of 83%	Fuses rs-fMRI and phenotype data; limited by age range (6–59 yrs) and generalization scope
[55]	ABIDE dataset	Site-adversarial DNN + ComBat-GAM	AUC: 0.70 ± 0.03	Tackled multi-site harmonization; struggled with convergence due to 36-site data complexity
[56]	EEG signals	CNN + XAI (LRP, PatternNet, Pattern-Attribution, Smooth-Grad Squared)	Not accuracy-based; ROAR for feature relevance	Highlights quantitative variability in XAI methods; evaluated interpretability on EEG emotion decoding
[57]	ASD data (Kaggle)	ML classifiers, best: AdaBoost	Accuracy: Not specified	Overfitting concerns: small dataset; emphasized need for larger, generalizable datasets
[58]	ABIDE + King Abdulaziz Univ. dataset	7 ML models + RFECV, Boruta + grey wolf optimizer (GWO) + SVM	Best: GWO + SVM (Accuracy: 71%)	Strong feature selection; accuracy may not meet clinical thresholds due to data heterogeneity

#### Highlights of neuroimaging analysis for ASD

Despite notable advances in neuroimaging and EEG-based technologies for the detection of ASD, a number of persistent research gaps continue to occur. The majority of studies utilize small and demographically restricted samples, which compromises the generalizability of results to more representative populations. Although promising, neuroimaging approaches are costly, equipment-based, and possibly challenging for young children with ASD because they are sensitive to noise and must be held still during scans. However, they play a central role in the generation of both research and clinical diagnostic development in ASD. The lack of gold-standard diagnostic validation tools like Autism Diagnostic Observation Schedule (ADOS) or Autism Diagnostic Interview – Revised (ADI-R) further curtails clinical usage. Most models are highly accurate but tend to be lacking in robustness for multi-site datasets, plagued with scanner and site variability. Explainability, while discussed in some research with SHAP, LIME, or Layer-Wise Relevance Propagation, is inconsistently adopted and frequently not clinician-validated. Additionally, few methods consider longitudinal or developmental paths, limiting observations into symptom course. Finally, although new models such as Graph Convolutional Network (GCNs) and federated architectures hold promise, they bring computational demands and frequently lack real-time capability, indicating scalable, interpretable, and clinically validated solutions are required.

### Genetic biomarkers

While ASD has historically been diagnosed by behavioral evaluation and developmental history, there has been increasing focus on incorporating genetic information to improve diagnostic accuracy. Several genes have been found to be linked with ASD, including variations in common and rare genetic changes. Genetic biomarkers, including single nucleotide polymorphisms (SNPs), copy number variations (CNVs), and patterns of DNA methylation, have been promising in differentiating ASD individuals from their typically developing counterparts. For example, mutations in genes such as SH3 and Multiple Ankyrin Repeat Domains 3 (SHANK3), Contactin-Associated Protein-Like 2 (CNTNAP2), and Chromodomain Helicase DNA Binding Protein 8 (CHD8) have been linked to ASD. Placental DNA methylation profiles have also been suggested as possible prenatal biomarkers for ASD [59].

Despite such progress, however, the use of genetic biomarkers in clinics is still in its infancy. Issues like heterogeneity of ASD, the availability of large numbers of diverse data sets, and the convergence of genetic information with other diagnostic technologies hold back their general use [60]. Still, continued research continues to investigate the ability of genetic biomarkers to deepen our knowledge and treatment of ASD. The following Table 4 shows current research done in this area.

**Table 4:** Work done using genomic data for ASD detection

References	Data source	AI/ML method	Outcome/Accuracy	Key insights/Limitations
[61]	5 GEO datasets; 709 blood-based samples	Meta-analysis + XGBoost + RFE + SHAP	46-gene subset identified; MID2 gene most influential	Highlighted MID2, AK3, RHOQ; limited clinical/genotype correlation; XAI only on GSE42133
[62]	NJLAGS cohort (111 families, 272 WGS)	Linkage + pVAAST + MetaSV + enrichment	207 high-confidence genes; loci on 12q & 17p	Neurodev. pathway enrichment; limited ADHD-WGS data; underrep. of non-coding variants
[63]	240 toddlers’ gene expression & DNA seq	42,840 models + BMA ensemble	AUC-ROC: 85–89%, AUC-PR: 84–92%	Robust across age, race; only male toddlers; clinical validation needed
[64]	AGP CNV + phenotype (2446 patients)	AHC + Naive Bayes + RF	NB precision: 0.82 (high IC), F1 overall: 0.279	Good for high-IC group; poor on full set; annotation gaps
[65]	Multi-omics + clinical data	iCluster, SNF, CNN, MKL	Not quantified (framework-focused)	Handles omics heterogeneity; no benchmark metrics
[66]	AGP CNV + phenotype (2446 patients)	AHC + Naive Bayes + RF	NB precision: 0.82 (high IC), F1 overall: 0.279	Good for high-IC group; poor on full set; annotation gaps
[67]	Spatiotemporal gene expression	ML-based gene ranking	Identified known/novel ASD genes	Prediction-based; not diagnostic
[68]	Genome-scale data	Ensemble ML (forecASD)	Outperformed earlier predictors	Focused on gene discovery; not for direct diagnosis

#### Highlights of genetic analysis for ASD

The use of genomic information for ASD prediction is an intricate and developing field of research. Although developments have been seen regarding the genetic causes of ASD, there still lies a formidable challenge in making predictive use of genetic information. The first and major challenge lies in the availability of extensive and high-quality genetic datasets. The rarity of large-scale, well-annotated genetic data sets puts many investigations in a state of limitation in being able to make firm conclusions or make trustworthy predictions regarding ASD from genetic data alone. Through studies, genetic investigations in ASD have revealed that several genes play a role in the causation of the disorder, but the specific genetic markers involved are still not clearly identified. This is also due in part to the fact that ASD is a heterogeneous disorder with a wide range of manifestations, and therefore it is hard to identify certain genetic variations that always relate to the disorder. Moreover, gene-environment interactions and environmental factors further complicate genetic predictions.

As a result of the limited amount of genetic data, the majority of current work on ASD detection has been confined to small sample sizes, which may limit generalizability. As such, prediction of ASD from genetic data alone is a poorly developed field, and more studies, including the acquisition of larger, more representative genetic datasets, are needed to enhance prediction models and reach more precise, personalized diagnostic tests. In summary, though genetic information has much potential for the detection and prediction of ASD, existing limitations, especially the absence of thorough and solid datasets, have limited the scope of available work.

### Eye gaze analysis

Eye gaze behavior has also been a promising non-invasive biomarker for the early identification of ASD. Since infancy, normal developmental pathways involve a preference for faces, mutual gaze, and joint attention—all of which are frequently impaired in children with ASD [69]. Studies have found that aberrant patterns of eye gaze, including decreased fixation on the eyes and reduced attention to social stimuli, are measurable as early as 6 months of age and are one of the earliest identifiable signs of ASD. Eye tracking methods provide the objective quantification of these behaviors with high temporal resolution and the possibility of measuring gaze behavior during naturalistic or screen-based conditions. When paired with ML algorithms, eye gaze data can be utilized with high precision to classify individuals with ASD, and thus is a potentially useful tool for early screening, particularly in preverbal or minimally verbal children. Eye gaze analysis, therefore, has great promise to create accessible, scalable, and interpretable diagnostic tools for early ASD intervention. The following Table 5 shows current research done in this area:

**Table 5:** Work done using eye gaze analysis for ASD detection

References	Data source	AI/ML method	Outcome/Accuracy	Key insights/Limitations
[70]	11 clinical ET + fNIRS studies, ages 3–17	SVM (fNIRS), metrics (ET)	ET: Prec 90%, Rec 69%, Spec 93%; fNIRS: 95.4%	Face fixation, background; device variability; small datasets
[71]	Kaggle (3014 images), ages 2–14	ConvNeXtBase + LightGBM	Accuracy: 95%, AUC: 0.91	Non-invasive; no clinical data; small dataset
[72]	86 children (30 TD, 56 ASD), video stimuli	Stacked ensemble (SVM, RF, etc.) + PCA	F1: 95.5%, Acc: 90.7%	Gaze anticipation features; interpretability reduced by ensemble complexity
[73]	Saliency4ASD (2033 pairs)	CNN (image + fixations) + LSTM + TracIn	Accuracy: 94.35%	Effective data distillation; limited dataset diversity
[74]	Kaggle + Zenodo, ages 2–14	Transfer learning + EAR + HOG + SVM	Accuracy: ~ 80%	EAR for attentiveness; limited by 2D RGB & non-clinical data
[75]	547 scan path images (59 children)	T-CNN-ASD vs traditional ML	Accuracy: 95.59%	No augmentation needed; small dataset, needs broader testing
[76]	547 ETSP images	PCA + DNN with augmentation	AUC: 97%, Sens: 93.28%	Strong results; overfitting risk with limited trials
[77]	34 children (Tobii eye tracker)	ACLNet + convLSTM + SVM	Accuracy: 68–100%	Fixation-based explainability; small, unaugmented sample
[78]	Saliency4ASD, 300 images, 5542 paths	STAR-FC + BrMLP, CNN + LSTM	AUC: 0.73, Accuracy: 62%	Scan-path useful but dataset small; future focus: explainability

#### Highlights of eye gaze analysis

In spite of increased interest in gaze analysis for ASD diagnosis, existing work shows significant shortcomings, mainly concerning the explainability and generalizability of ML models. Whereas some studies are finding high classification accuracy with DL and ensemble models (e.g., T-CNN-ASD, stacked ensemble, CNN-LSTM hybrids), most are without comprehensive explainability frameworks that can explain predictions transparently to clinicians. Few works, including those using TracIn or fixation-based interpretation (e.g., ACLNet + convLSTM), try to offer such insights into model decisions. Yet, these approaches tend to be limited by small and demographically restricted datasets, a lack of real-time interpretability, or technical complexity that prevents clinical adoption. In addition, most works base their conclusions on pre-processed image or scan-path data, which does not include context-specific interpretation of gaze behavior in naturalistic environments. Therefore, there is an urgent need for creating explainable, clinically interpretable, and scalable eye gaze-based models to bridge the performance gap between high-performance AI and actual autism screening practice.

## Comparative analysis

**Fig. 5:**
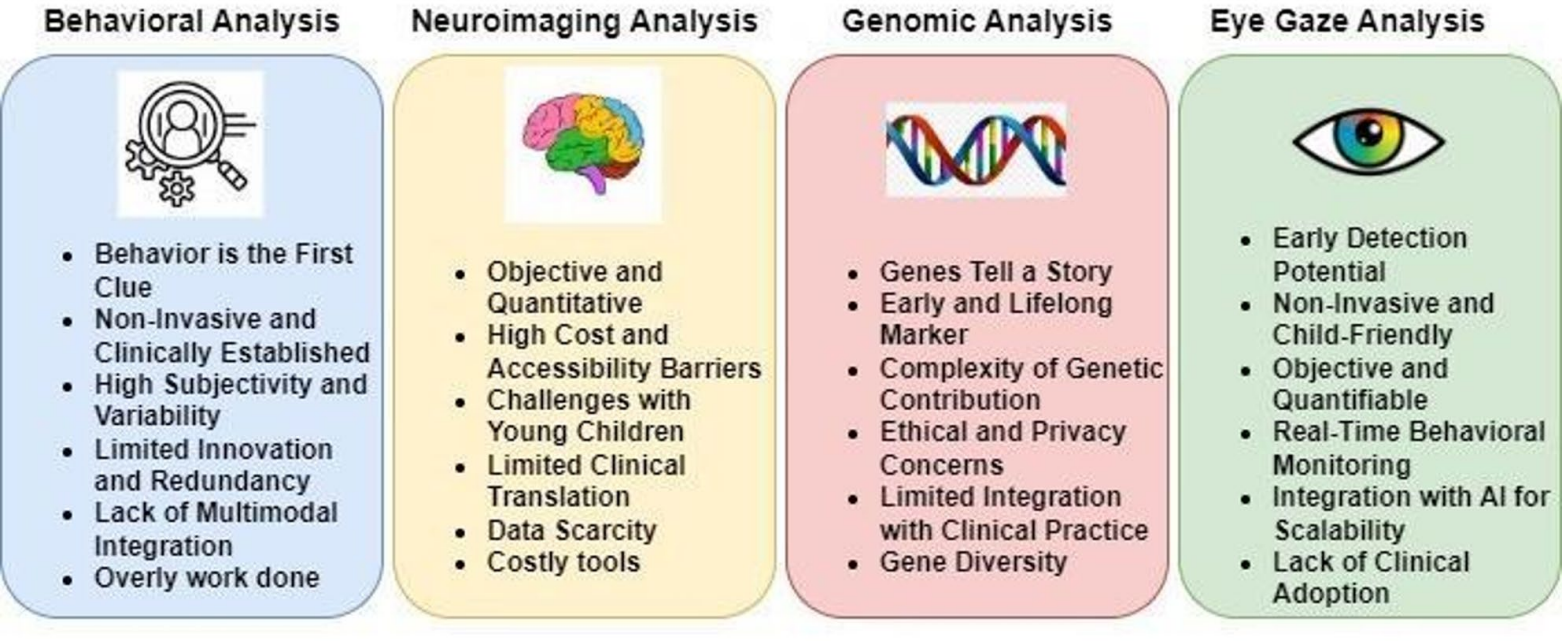
Comparative analysis of four biomarkers

Table 6 presents a comparative analysis of the four primary biomarker categories—behavioral, neuroimaging, genetic, and eye gaze—evaluated across key parameters such as cost-effectiveness, feasibility, diagnostic accuracy, interpretability, and clinical applicability. This comparison highlights the strengths and limitations of each biomarker type, offering a consolidated view to guide future research and clinical implementation strategies in early ASD detection. Figure 5 gives a visual representation of the same.

**Table 6:** Comparative analysis of biomarkers

Comparison parameter	Cost-effective	Non-invasive	Clinical feasibility	Data availability	Explainability potential	Standardization	Scalability	Data objectivity	Cross-cultural	Automation readiness
Behavioral	✓	✓	✓	✓	✓					
Neuroimaging					✓			✓		
Genomic					✓			✓		
Eye gaze	✓	✓	✓	✓	✓	✓	✓	✓	✓	✓

## Role of XAI in autism detection

### XAI in healthcare

Explainable Artificial Intelligence (XAI) has evolved as an essential discipline to bridge the inherent "black-box" quality of sophisticated AI models by delivering comprehensible and transparent information about their decision-making processes. As noted by [18], XAI frameworks are developed under four broad categories: data explainability, model explainability, post-hoc explainability, and explanation evaluation. These elements individually target various phases of the AI pipeline to foster transparency across developers, domain experts, and end-users. The research recommends that equally weighing model interpretability with prediction accuracy is key to wider acceptance and clinical incorporation of AI systems, calling for future studies to prioritize human-centered design and domain-specific tailoring of explanations. XAI studies aim to construct models that satisfy high performance requirements while being explicable to human beings. The development of XAI methods has been driven, according to new surveys, by increasing industrial, regulatory, and societal needs with efforts like DARPA's XAI initiative and greater deployment by firms such as Microsoft and H2O.ai. In spite of these developments, the area still has challenges involving standardizing metrics for evaluation, adapting explanations to various user groups, and balancing model accuracy versus interpretability, indicating that XAI will continue to be an important and developing field of AI research [19]

XAI is increasingly seen as an essential part in building reliable AI systems, especially in high-stakes domains like healthcare. While DL models provide excellent predictive accuracy, their interpretability is restricted, and hence, their clinical adoption and trustworthiness are curbed. Existing XAI techniques, including attribution-based methods (e.g., SHAP, Grad-CAM) and concept-based explanations (e.g., TCAV), have contributed a great deal to model transparency. Despite this, challenges still exist, such as the lack of standardized metrics for evaluation and the difficulty in explaining multimodal medical data. As emphasized by Holzinger et al. [79], obtaining genuinely explainable AI will involve overcoming these limitations to facilitate transparency, fairness, and reproducibility in medical use cases, leading the way towards more responsible and accepted AI solutions in clinical use.

Some of the key applications of XAI in healthcare are- as in [80], scientists have created an XAI tool that can forecast side effects such as lymphoedema in breast cancer patients three years after treatment. The model was trained on information from more than 6,300 patients and recorded a 73.4% accuracy rate, giving clinicians easily interpretable results, which can inform personalized treatment planning. XAI techniques, such as Grad-CAM, assist radiologists in visualizing key features in medical images, enhancing interpretability and reducing misdiagnoses. XAI accelerates drug discovery by analyzing vast datasets and providing clear explanations for AI-generated recommendations, aiding researchers in identifying potential drug candidates. XAI enhances CDSS by offering transparent explanations for AI-generated recommendations, supporting informed decision-making among healthcare professionals [81]. XAI improves remote patient monitoring by analyzing data from wearables and sensors, providing insights into patient health trends and supporting early intervention strategies. A systematic review analyzed 68 studies on XAI in Clinical Decision Support Systems (CDSS), highlighting advancements and identifying challenges. The review emphasizes the need for more public datasets, advanced data treatment methods, and comprehensive evaluations of XAI methods to enhance usability and effectiveness in clinical settings [82].

### Significance of explainability in ASD detection

Explainability is imperative ASD identification because it fills the gap between sophisticated AI systems and clinical decision-making. Because ASD diagnosis entails very individualized and heterogeneous behavioral and biological profiles, interpretable AI systems enable clinicians and caregivers to see why a specific diagnosis or risk forecast is given. Explainable models not only increase trust by demonstrating which characteristics—like communication deficits, stereotyped behavior, or neuroimaging signatures—are driving decisions but also enable earlier and more individualized interventions. Explainability is also necessary for ethical and regulatory purposes, can be used to identify possible biases in populations, and may lead to greater scientific insight into risk factors and ASD subtypes. Therefore, incorporating explainability into AI models is essential to make them responsibly used, unbiased, and effective in the diagnosis and treatment of autism.

In addition, explainability aids in creating more fair AI systems by allowing biases and errors to be revealed. ASD usually manifests differently among genders, ethnicities, and age groups, and opaque models threaten to entrench current differences. With explainable methods such as SHAP or LIME, researchers and clinicians can evaluate feature contributions and refine models for more balanced, generalized performance. This is especially relevant due to recent under diagnosis in girls and minorities, where more atypical presentation may be missed with traditional criteria.

Finally, explainability is essential to continuing research and innovation in autism science. By identifying which neuroanatomical, genetic, or behavioral characteristics regularly affect AI predictions, scientists can create new hypotheses regarding ASD's underlying mechanisms. This sets up a feedback loop in which explainable AI not only helps in clinical diagnosis but also inspires deeper scientific investigation, ultimately contributing to improved understanding, earlier diagnosis, and more effective interventions for people across the autism spectrum.

### Popular XAI methods used in ASD research

Table 7 gives an organized comparison of some of the widely used Explainable AI (XAI) methods used in ASD detection. The table gives major features of a technique, such as identifying if it is gradient-based or perturbation-based, model-specific or model-agnostic, local or global, and whether explainability is inherent to the model or added post hoc. Each technique has a concise description and the usual applications in ASD research, varying from feature attribution to neuroimaging to saliency detection to eye-tracking and behavioral data. The overview facilitates the choice of suitable XAI techniques by researchers and clinicians according to model type, interpretability requirements, and clinical significance.

**Table 7:** XAI methods used in ASD research

Technique name	References	Gradient/Perturbation	Model specific/Model agnostic	Local/Global	Intrinsic/Post-Hoc	Brief description	Typical application in ASD detection
LIME	[83–86]	Perturbation	Model Agnostic	Local	Post Hoc	Local surrogate models for interpretability	Behavioral screening, Eye gaze analysis
SHAP	[87–89]	Perturbation	Model Agnostic	Both	Post Hoc	Feature importance via Shapley values	Genomics, Neuroimaging, Behavioral data
LRP	[90]	Gradient	Model Specific	Local	Intrinsic	Backpropagation of relevance scores	EEG-based ASD classification
CAM	[91, 92]	Gradient	Model Specific	Local	Post Hoc	Highlights spatial regions in input data	Neuroimaging or eye-tracking
Grad-CAM	[93]	Gradient	Model Specific	Local	Post Hoc	Class activation mapping for CNNs	Neuroimaging, Eye-tracking
Saliency map	[94–97]	Gradient	Model Specific	Local	Post Hoc	Highlights key input regions influencing predictions	Neuroimaging, facial, or eye-tracking data

### Applications across biomarker domains

**Fig. 6:**
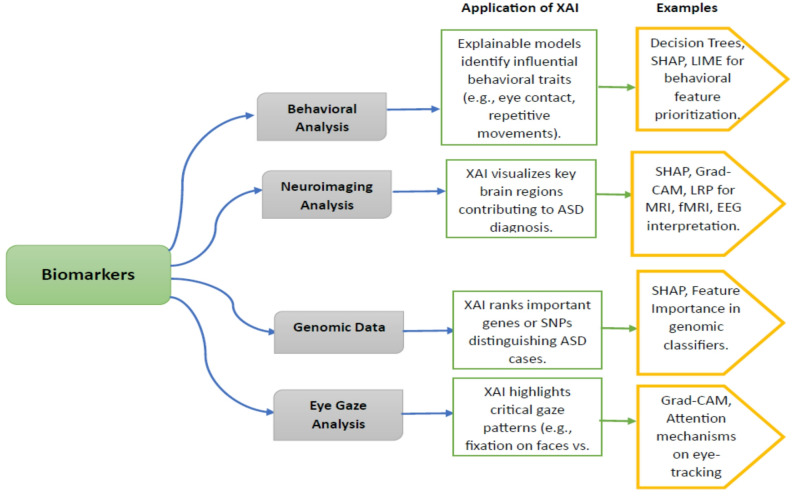
Biomarker based applications of XAI methods

Figure 6 summarizes how XAI techniques have been used in all four principal biomarker types—behavioral, neuroimaging, genetic, and eye gaze within the context of detecting ASD. Each of these domains has particular data type, dimensionality, and interpretability issues that make the use of XAI techniques highly beneficial. From detecting significant gaze features in eye-tracking measurements to visualizing the patterns of neural activation in neuroimaging, XAI fosters explainability and facilitates clinical validation. The subsequent analysis illustrates the increasing synergy between domain-specific biomarkers and explainability frameworks, the road to more reliable and actionable diagnostic models.

### Benefits of XAI in ASD research

- *Increases trust and transparency*

XAI models explain how and why predictions are made, making clinicians, researchers, and caregivers more confident in using AI-driven ASD diagnostic tools [98].

- *Enhances clinical Interpretability*

By showing which features (like gaze behavior, brain regions, or genetic markers) drive predictions, XAI bridges the gap between complex AI models and human clinical reasoning [99].

- *Supports early and accurate diagnosis*

XAI helps in identifying subtle patterns in multimodal data, enabling earlier detection and potentially improving long-term outcomes through timely interventions [100].

- *Facilitates model validation and debugging*

XAI allows researchers to check if models are making decisions based on meaningful, biologically or behaviorally relevant features—thus improving model reliability [101].

- *Improves reproducibility and generalizability*

Transparent AI models are easier to validate across different datasets and settings, leading to more robust, generalizable solutions for ASD detection [102].

- *Encourages ethical and responsible AI use*

Explainable models align with ethical standards and regulatory requirements, ensuring fairness, reducing bias, and enabling informed consent in clinical applications [103].

- *Promotes integration into clinical workflows*

Because clinicians need understandable outputs for diagnosis and decision-making, XAI makes AI tools more practical and acceptable for real-world use [104].

- *Electronic health records*

XAI is crucial in improving the interpretability and trustworthiness of machine learning models used in Electronic Health Records (EHRs). EHRs hold enormous, heterogeneous patient data ranging from demographics and diagnoses to medications and clinical notes, making them worth using for predictive analytics in healthcare [105].

### Clinical translation pathway

A systematic approach that connects technological advancement with real-world implementation is necessary for the effective clinical translation of AI and XAI-based ASD detection frameworks. This involves adhering to ethical and regulatory standards, enhancing model interpretability to foster clinician trust, and ensuring that data consistency and multi-site validation considering population diversity. Integration also requires pilot studies for deployment in actual clinical workflows and integration with electronic health records (EHRs). Establishing standardized procedures for data collection, model evaluation, and interpretability reporting also requires cooperation between data scientists, clinicians, and lawmakers as well. In order to evaluate explainable models' stability and dependability across various age groups and developmental stages, it is crucial that they go through longitudinal validation. Clinicians may find it easier to understand model outputs if decision-support tools and user-friendly visual dashboards are included. Resolving these translational issues can accelerate the shift from experimental models to clinically useful diagnostic instruments, ultimately promoting accurate and timely identification of ASD and enhancing patient outcomes globally.

## Challenges and limitations


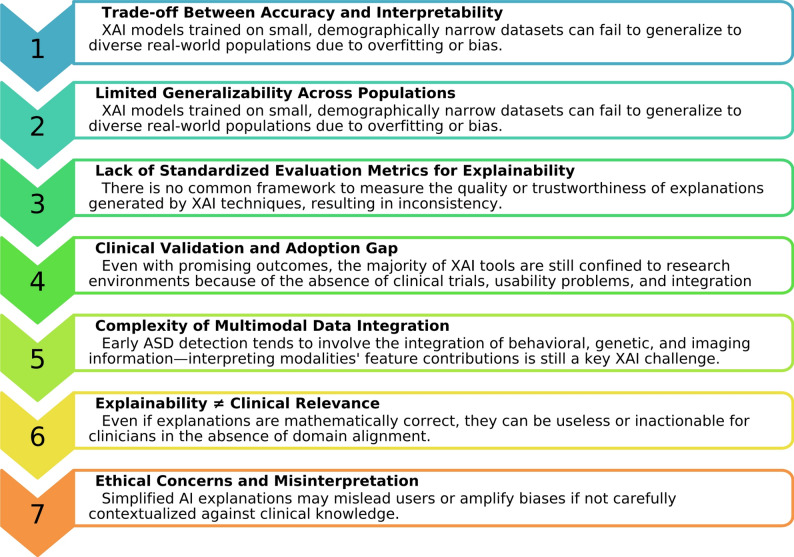


## Future directions

Of the four large-scale biomarkers investigated for detecting ASD-behavioral patterns, neuroimaging, genomic information, and eye gaze analysis, behavioral analysis has already been the subject of widespread investigation with well-established techniques. Although neuroimaging and genomic methods are of high biological validity, practical implementation is generally hampered by high expense, ethical issues, and limited availability in actual clinical practice. Second, while the incorporation of XAI in these fields is still limited, it is frequently because of the nature of the data and poor interpretability to clinicians. Eye gaze analysis is a promising, non-invasive, and scalable solution that measures early behavioral indicators of ASD. The main focus of our future research will be developing multi-modal eye-gaze datasets that capture both temporal and spatial gaze dynamics in order to create more reliable and broadly applicable models. In order to provide both local and global interpretability of predictions and aid in clinician comprehension, we intend to deploy hybrid explainable AI frameworks (e.g., combining SHAP, LIME, and Grad-CAM). To assess the clinical utility, reliability, and interpretability of these models, we also hope to carry out validation studies with clinicians in the loop. Additional measures to guarantee model scalability include demographic diversification and cross-site testing. Through these efforts, the research seeks to develop XAI-supported eye gaze-based ASD detection systems that are clinically meaningful, transparent, and deployable.

## Conclusion

Early and accurate diagnosis of ASD is essential for early intervention and enhanced developmental outcomes. This review analyzed four broad biomarker domains—behavioral, neuroimaging, genomic, and eye gaze data—and how they are combined with machine learning and explainable AI methods. Although behavioral analysis is the most studied domain, its potential for further innovation is limited. Neuroimaging and genomic biomarkers, rich with biology though they are, have challenges like high expense, ethical limitations, and narrow interpretability. Explainable AI techniques such as SHAP, LIME, and CAM have improved model transparency in numerous domains, but their incorporation remains an underexploited asset in complex clinical datasets. With these observations, eye gaze analysis appears to be an extremely promising avenue, with non-invasive, objective, and scalable attributes being particularly well-suited to early ASD screening. Combining eye-tracking information with XAI techniques has promise for creating explainable, interpretable, and deployable solutions that will close the gap between clinical practice and AI research.

## Data Availability

No datasets were generated or analysed during the current study.
